# Quantitative analysis of carnosine, anserine, and homocarnosine in skeletal muscle of aquatic species from east China sea

**DOI:** 10.1016/j.bbrep.2020.100880

**Published:** 2020-12-22

**Authors:** Chun-yue Wang, Yan-rong Li, Chen Pan, Jian Chen, Wei Jiang, Wei-nan Li, Xiao-lin Zhang, Zhi Liao, Xiao-jun Yan

**Affiliations:** a Laboratory of Marine Biology Protein Engineering, Marine Science and Technical College, Zhejiang Ocean University, Zhoushan City, 316022, Zhejiang, China; b Ningbo Institute of Oceanography, Ningbo City, 315800, Zhejiang, China

**Keywords:** Histidine-containing dipeptide, Carnosine, Anserine, Homocarnosine, Aquatic species

## Abstract

Histidine-containing dipeptides (HCDs) are a family of non-protein, nitrogen-containing compounds with multiple physiological roles and are mainly present in excitable tissues of vertebrates. The distribution of HCDs in various animal species has been the subject of study for nearly 100 years. The aim of this research was to determine the content of the HCDs in the aquatic species collected from the Zhoushan fishing ground of the East China Sea. Using LC-MS/MS technology, the occurrence of carnosine, anserine, and homocarnosine in skeletal muscle of 38 aquatic species (26 teleosts, 6 molluscs, and 6 crustaceans) and chicken breast was investigated. Of the 38 aquatic species examined, 24 species (23 teleosts and 1 mollusc) contained considerable amounts (>5 ng/g wet tissue) of HCDs, and anserine was the major component of HCDs in their skeletal muscles. Only 5 teleosts contained homocarnosine. Most invertebrates, with the exception of the sepia *Uroteuthis chinensis*, did not contain HCDs. The present findings greatly expand the HCD distribution data and provide insight into understanding the roles of HCDs in different animals and a nutritional assessment for marine aquatic species.

## Highlights

• carnosine, anserine, and homocarnosine were determined in 38 marine species.
• Almost all the tested fish contain histidine-containing dipeptides.
• The highest value was presented in migratory pelagic fishes.
• The anserine is the major component in marine species.
• No HCD can be detected in invertebrates with the exception of the family Loliginidae.

## 1 Introduction

Carnosine (β-alanyl-l-histidine) is a natural dipeptide that was first discovered in 1900 by Vladimir Gulevitch as an abundant non-protein, nitrogen-containing compound of meat. Carnosine is an archetype of a family of histidine-containing dipeptides (HCDs), and several members of this family have been identified subsequently [1], including anserine (β-alanyl-Nπ-methyl-l-histidine), balenine (also called ophidine, β-alanyl-Nτ-methyl-l-histidine), and homocarnosine (γ-aminobutyryl-l-histidine). Recent findings have highlighted the important roles of HCDs in muscular function and homeostasis, including their pH buffering ability, antioxidant capacity, increased Ca^2+^ sensitivity and protein glycation inhibition [2,3]. The highest concentration of HCDs is observed in skeletal muscle, cardiac muscle, the brain and olfactory bulb, the stomach, and the kidneys of vertebrates [4]. However, the biological role of carnosine and its analogues is still not fully understood.

The distribution pattern of HCDs in various animal species has been the subject of study for nearly 100 years. Carnosine and its analogues are mainly present in vertebrates, including mammals, fishes, amphibians, birds, and reptiles [2]. Nobody has ever detected them in invertebrates or other eukaryotes, such as plants and fungi, for now. On the other hand, the data available on the quantitative analysis of HCDs are focused on carnosine and its methylated derivatives (anserine and ophidine/balenine), and the quantitative analysis of homocarnosine in animals is very limited. Homocarnosine is a brain specific dipeptide of γ-aminobutyric acid (GABA) and l-histidine [5] in the mammalian central nervous system [6]. Literature on the distribution of homocarnosine in aquatic species is rare.

The Zhoushan fishing ground of the East China Sea is a world-famous fishery with rich biodiversity. Considering the important roles of HCDs in physiology and nutrition, it is necessary to explore the HCD contents in different aquatic species of the Zhoushan fishing ground. To date, the available methods for quantifying carnosine include the *o*-phthalic aldehyde method (OPA) [7], high-performance liquid chromatography (HPLC) [8], electrophoresis [9], liquid chromatography–mass spectrometry (LC-MS) analysis [10], and nuclear magnetic resonance (NMR) [11]. LC-MS has been concluded to be rapid, sensitive and selective for the determination of HCDs in animal tissues [12]. Therefore, based on ultra-performance liquid chromatography tandem mass spectrometry (UPLC-MS/MS), the contents of carnosine and its analogues (anserine and homocarnosine) were determined simultaneously in muscle tissue from 36 different aquatic species, including 24 teleosts, 6 molluscs, and 6 crustaceans. In addition, two tuna, one salmon, and chicken breast were used as controls. In the present study, we found that almost all teleosts and only one mollusc (*Uroteuthis chinensis*) contain one or more carnosine analogues, and no HCD was detected in the crustaceans. In most cases, the methylated form is more abundant than nonmethylated carnosine. The results of the present study greatly expanded the HCD distribution data and provided a clue for understand the roles of HCDs in different species.

## 2 Materials and methods

### 2.1 Chemicals

l-Carnosine was obtained from Sigma-Aldrich (Shanghai, China). l-anserine and l-homocarnosine were synthesized by ChinaPeptides™ (Shanghai, China). HPLC grade water was obtained from a MilliQ system. HPLC grade acetonitrile was obtained from TEDIA (Fairfield, USA). Standard solutions were prepared using a starting eluent, acetonitrile/water (1:1, v/v) containing 0.1% formic acid, as a solvent at six concentration levels (10, 12.5, 25, 50, 100, and 200 μg/L).

### 2.2 Sample preparation

Chicken breast, tuna (*Thunnus tonggol* and *Katsuwonus pelamis*) and trout (*Oncorhynchus aguabonita*) were purchased from a local supermarket. Twenty-three marine fish species, six molluscs, and six crustaceans were caught during the summer from the East China Sea near Zhoushan Islands by fishing vessels and were immediately frozen at −20 °C. Species identification were performed by morphological characteristics (including body shape, scale, fin type, teeth and skeleton, etc.) for fish, shell number and shell morphologies (such as spire, body whorl, growth lines, arm, sucker, etc.) for mollusc, and carapace morphologies (such as pereiopodsand, abdominal limb, etc.) for crustaceans. The database of World Register of Marine Species (WoRMS, http://www.marinespecies.org/) and Fishbase (https://www.fishbase.in/) were also used for species identification.

The skeletal muscle (~1 g) of each species was collected from three individual specimens, weighed, and smashed immediately using liquid nitrogen. The smashed tissue was then homogenized in pure water by a handheld homogenizer. The homogenate was further treated by an ultrasonic cell crusher to release intracellular HCDs. The samples were then deproteinized by adding 10% trichloroacetic acid and centrifuged at 20,000×*g* for 5 min at 4 °C. The supernatants were ultra-filtrated by a 3 kD cutoff, and the filtered solution was transferred to vials and lyophilized for use.

### 2.3 LC-MS/MS analysis

Chromatographic separation was performed on a reverse phase Acquity UPLC BEH amide column (1.7 μm, Waters) using a Thermo Fisher U3000 High Performance Liquid Chromatography (HPLC) system. Elution was conducted using acetonitrile/water (3:7, v/v) as mobile phase A and acetonitrile/water (8:2, v/v) as mobile phase B, which were introduced with a flow rate of 0.15 mL/min. Then, 0.1% ammonium hydroxide was added to the mobile phases as an additive. Gradient elution was programmed as follows: 0–10 min, 100 - 60% B; 10–11 min, 60–40% B; 11–11.5 min, 40–100% B; 11.5–20 min, 100% B. The temperature of the sample chamber was set at 8 °C, and the column temperature was set at 35 °C. The column was equilibrated for 10 min at initial conditions.

Mass spectrometry (MS) was performed on a Thermo Scientific™ Q-Exactive hybrid quadrupole-Orbitrap mass spectrometer equipped with a HESI-II probe. The instrument was operated using a full MS method in positive modes. Data were acquired from 150 to 500 *m*/*z* at 70 K resolution using centroid mode. The AGC target was set as 3e6 for MS. The capillary voltage was set at 3.5 kV, and the capillary temperature was 275 °C.The sheath gas was 30 arb, and the aux gas was 10 arb. The instrument was previously calibrated in positive modes.

## 3 Results and discussion

### 3.1 Chromatographic analysis

**Fig. 1:**
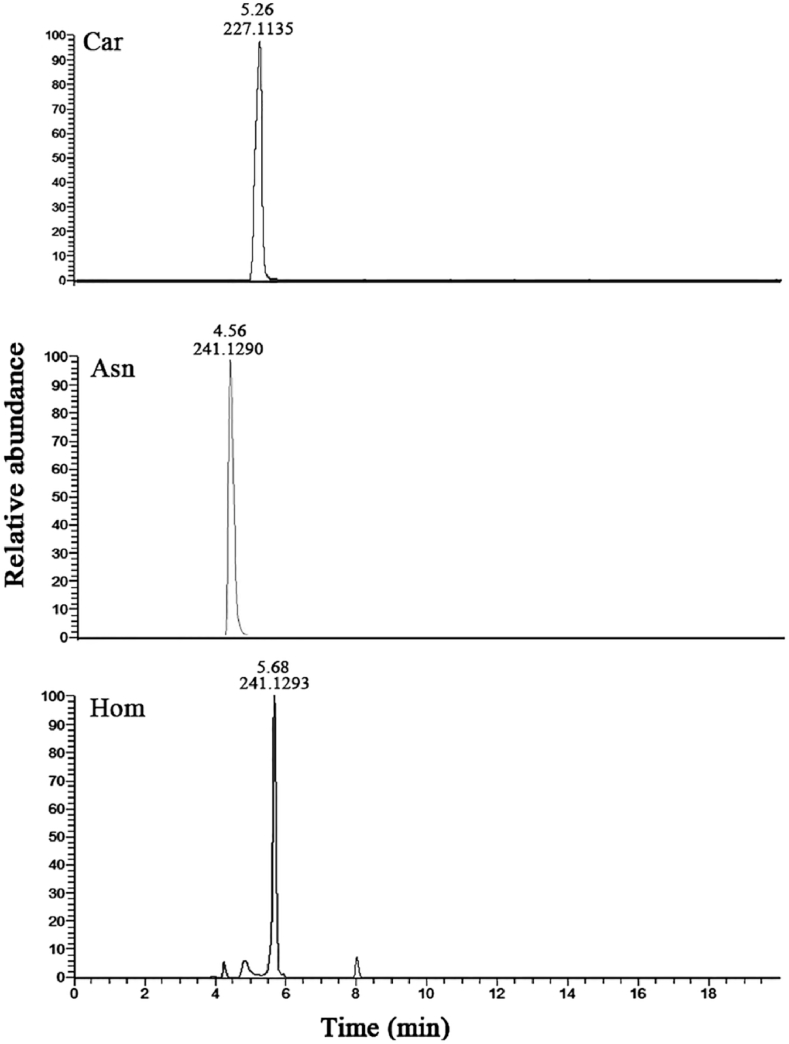
MS chromatogram of the carnosine (Car, 227.1135 m/z), anserine (Ans, 241.1290 *m/z*), and homocarnosine (Hom, 241.1294 *m/z*) eluted on a reverse phase Acquity UPLC BEH Amide column using a Thermo Fisher U3000 High Performance LC system.

Carnosine, anserine, and homocarnosine were identified in the specimens based on preliminary separation/fragmentation study and quantified in different aquatic species using calibration curves obtained from the standard carnosine and its analogues. A representative chromatogram of carnosine and its analogue separation is shown in Fig. 1. The protonated molecular ions were 227.11 *m*/*z* for carnosine and 241.13 *m*/*z* for both anserine and homocarnosine. Although anserine and homocarnosine have the same *m*/*z*, the different structures of the two molecules gave different MS/MS spectra, which can be therefore used to discriminate between these two molecules. The detection limits (according to the S/N = 3 criterion) were 10 ng/mL for carnosin and its analogues. Carnosin, anserine, and homocarnosine were quantified in the range of 50 ng/mL to 10 μg/mL. The correlation coefficient (R^2^) of the calibration curve was 0.9923 for carnosin, 0.9995 for anserine, and 0.9926 for homocarnosine. The intra- and inter-day accuracy values of carnosine related compounds were within ±2.2% and ±14.6%, respectively. These results indicated that the established method for quantification of carnosine related compounds is highly accurate and precise.

### 3.2 Interpretation of MS data

**Fig. 2:**
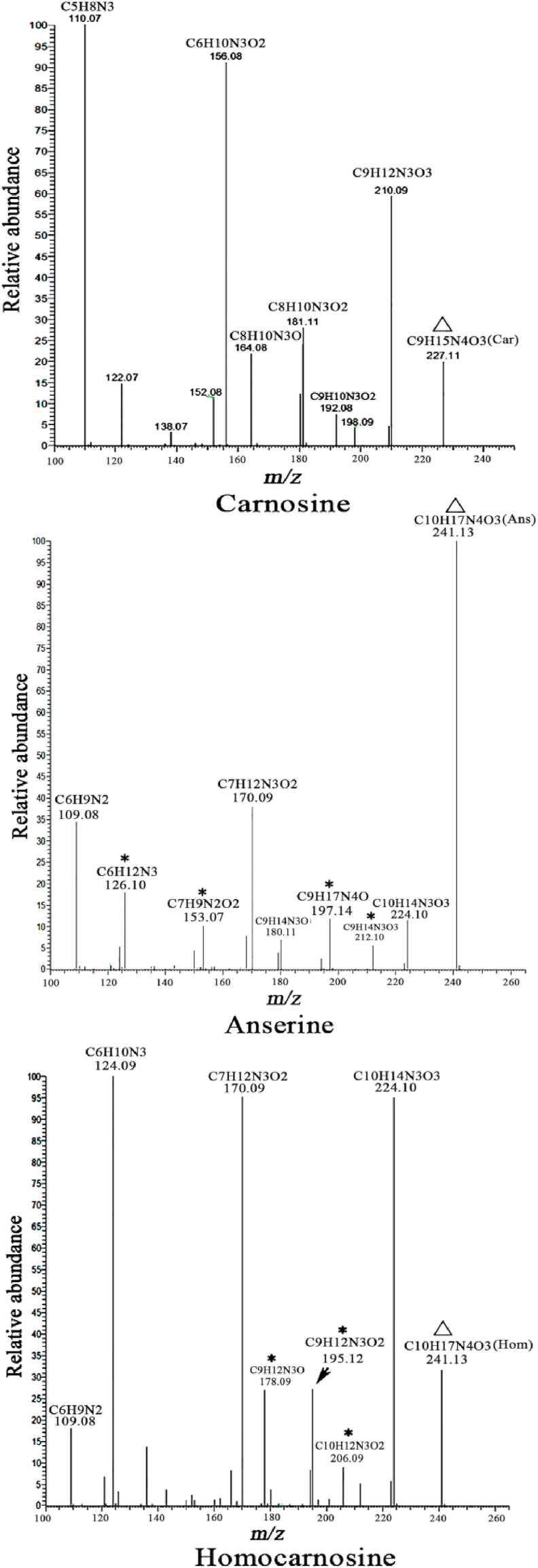
MS/MS spectrum of protonated ion of the carnosine (Car, 227.1135 *m/z*), anserine (Ans, 241.1290 *m/z*), and homocarnosine (Hom, 241.1294 *m/z*) isobaric compounds. The triangle represent the precursor ion, and the stars represent the specialized *m/z* for discriminating anserine and homocarnosine.

Fig. 2 shows the MS/MS spectra and the relative fragmentation interpretation of carnosine, anserine, and homocarnosine. The relative molecular formula of fragments can be interpreted according to the methods provided by Peiretti et al. and confirmed by MS^3^ analysis [10]. In the MS/MS spectrum of carnosine (Fig. 2), peaks with *m/z* of 210.09 (C_9_H_12_N_3_O_3_), 164.08 (C_8_H_10_N_3_O), 156.08 (C_6_H_10_N_3_O_2_), and 110.07 (C_5_H_8_N_3_) were detected as the most abundant fragment peaks from the precursor ion (C_9_H_15_N_4_O_3_). Anserine and homocarnosine presented an almost identical *m/z* of 241.13 in the different MS/MS spectra (Fig. 2). In the MS/MS spectrum of anserine, peaks with *m/z* of 224.10 (C_10_H_14_N_3_O_3_), 197.14 (C_9_H_17_N_4_O), 170.09 (C_7_H_12_N_3_O_2_), 126.10 (C_6_H_12_N_3_), and 109.08 (C_6_H_9_N_2_) were detected as the most intense fragment peaks from the precursor ion (C_10_H_17_N_4_O_3_). For the MS/MS spectrum of homocarnosine, peaks with *m/z* of 224.10 (C_10_H_14_N_3_O_3_), 195.12 (C_9_H_12_N_3_O_2_), 178.09 (C_9_H_12_N_3_O), 170.09 (C_7_H_12_N_3_O_2_), 124.08 (C_6_H_10_N_3_), and 109.08 (C_6_H_9_N_2_) were the most intense fragment peaks from the precursor ion (C_10_H_17_N_4_O_3_). The homocarnosine MS/MS spectrum shows a pattern similar to that of anserine. However, some fragment peaks are different between the two isomers, such as the peaks with *m/z* of 126.10, 153.06, 197.14, and 212.10 for anserine and 178.09, 195.12, and 206.09 for homocarnosine (Fig. 2).

### 3.3 Quantification of carnosine, anserine, and homocarnosine in different species

The final contents (μg/g wet tissue) of HCDs (carnosine, anserine and homocarnosine) in the muscle of each species are shown in Table 1. In this study, the HCD contents of chicken breast, tuna, and trout are comparable with previous studies. Chicken breast has the highest HCD concentration of ~7 μg/g carnosine and ~21 μg/g anserine. These concentrations are comparable with results (~8 μg/g carnosine and ~15 μg/g anserine) reported previously [10]. Considering the fact that even in the same species, the HCD contents differ according to the part of the animal, the process model of the sample, and the sex, age, and genotype of the specimen [13], the difference in the HCD contents in chicken breast between this work and other reports is acceptable. In addition, our results for tuna (*Thunnus tonggol* and *Katsuwonus pelamis*) and trout (*Oncorhynchus aguabonita*) are also in the range of values obtained in previous studies [2,[14], [15], [16]], indicating the reliability of the method for the quantification of HCDs in this study and the greater abundance of HCDs in migratory fishes [14].

**Table 1:** Carnosine, anserine and homocarnosine contents (μg/g wet tissue) in skeletal muscle obtained from different species (triple extraction, means ± SD).

No.	Family	Species	Carnosine (μg/g)	Anserine (μg/g)	Homocarnosine (μg/g)	Ratio of anserine/carnosine
**Birds**	Phasianidae	*Gallus domesticus*	6.968 ± 0.187	21.105 ± 0.452	–	3.0
**Telostei**						
1	Scombridae	*Thunnus tonggol*	0.014 ± 0.002	7.061 ± 0.896	–	504.4
2		*Katsuwonus pelamis*	1.117 ± 0.147	4.665 ± 0.382	–	4.2
3		*Scomber japonicus*	–	–	0.150 ± 0.072	
4	Sciaenidae	*Larimichthys polyactis*	0.020 ± 0.006	0.030 ± 0.010	0.002 ± 0.001	1.5
5		*Larimichthys crocea*	0.007 ± 0.0002	0.454 ± 0.014	–	64.9
6		*Pennahia argentata*	–	0.079 ± 0.001	–	
7		*Miichthys miiuy*	–	0.025 ± 0.0006	–	
8	Trichiuridae	*Trichiurus lepturus*	0.002 ± 0.0003	0.130 ± 0.025	0.050 ± 0.013	65.0
9	Malacanthidae	*Branchiostegus japonicus*		0.675 ± 0.005	–	
10	Stromateidae	*Pampus argenteus*	0.010 ± 0.003	0.497 ± 0.104	–	49.7
11	Priacanthidae	*Priacanthus macracanthus*	0.022 ± 0.0009	4.129 ± 0.778	–	187.7
12	Sparidae	*Acanthopagrus schlegelii*	0.0004 ± 0.0005	0.120 ± 0.019	–	300.0
13	Serranidae	*Lateolabrax japonicus*	–	0.025 ± 0.002	–	
14	Terapontidae	*Terapon theraps*	–	0.036 ± 0.008	–	
15	Sphyraenidae	*Sphyraena japonica*	–	0.999 ± 0.063	–	
16	Engraulidae	*Setipinna taty*	–	0.059 ± 0.011	–	
17	Centrolophidae	*Psenopsis anomala*	–	–	–	
18	Carangidae	*Trachurus japonicus*	–	0.494 ± 0.060	–	
19	Gobiidae	*Boleophthalmus pectinirostris*	–	–	–	
20	Antennariidae	*Antennarius striatus*	–	–	–	
21	Salmonidae	*Oncorhynchus aguabonita*	0.019 ± 0.002	3.082 ± 0.224	–	162.2
22	Scomberesocidae	*Cololabis saira*		0.027 ± 0.004	–	
23	Synodontidae	*Harpadon nehereus*	–	–	0.007 ± 0.0008	
24	Cynoglossidae	*Cynoglossus abbreviatus*	–	0.051 ± 0.021	–	
25	Monacanthidae	*Thamnaconus modestus*	–	0.006 ± 0.001	–	
26	Congridae	*Conger myriaster*	–	–	0.491 ± 0.069	
**Mollusks**						
1	Melongenidae	*Hemifusus tuba*	–	–	–	
2	Fasciolariidae	*Fusinus colus*	–	–	–	
3	Bursidae	*Bufonaria rana*	–	–	–	
4	Loliginidae	*Uroteuthis chinensis*	0.003 ± 0.0007	0.015 ± 0.005	–	5.0
5	Sepiidae	*Epiella japonica*	–	–	–	
6	Mytilidae	*Mytilus coruscus*	–	–	–	
**Crustaceans**						
1	Penaeidae	*Parapenaeopsis hardwickii*	–	–	–	
2	Solenoceridae	*Solenocera melantho*	–	–	–	
3	Squillidae	*Oratosquilla oratoria*	–	–	–	
4	Goneplacidae	*Carcinoplax longimana*	–	–	–	
5	Calappidae	*Calappa philargius*	–	–	–	
6	Portunidae	*Charybdis feriata*	–	–	–	

For the 26 tested fishes in this study (Table 1), there is a large range of total HCD concentrations in different species, with some of the highest values (>3 μg/g) presented in the migratory fishes, such as tuna and trout, and lowest values (<0.03 μg/g) presented in *Thamnaconus modestus*, *Miichthys miiuy*, and *Lateolabrax japonicus*. Of the 3 species of the family *Scombridae* belonging to the order *Perciformes*, the tuna *Thunnus tonggol* contained the highest level (7.075 μg/g) of HCDs in its skeletal muscle, followed by *Katsuwonus pelamis* (5.782 μg/g). HCD was also detected at low levels (0.15 μg/g) in caballa *Scomber japonicas*. Of the 4 species of the family *Sciaenidae* in the same order, *Larimichthys crocea* (big yellow fish) contained relatively high HCD concentration of 0.461 μg/g tissue, and three other *Sciaenidae* species contained < 0.1 μg/g HCD. For other species of *Perciformes*, the highest concentration of HCD was presented in *Priacanthus macracanthus* (4.151 μg/g), followed by *Sphyraena japonica* (0.999 μg/g), *Branchiostegus japonicas* (0.675 μg/g), and *Pampus argenteus* (0.507 μg/g). No HCD was detected in *Boleophthalmus pectinirostris* and *Psenopsis anomala*. For other orders except *Perciformes*, the HCD content is very variable. A high level (3.101 μg/g) of HCD was detected in the skeletal muscle of *O. aguabonita*, followed by *Conger myriaster* (0.491 μg/g). The fishes from other orders contained a very low level (<0.1 μg/g) of HCD.

According to the present data, no obvious rule can be concluded for the distribution pattern of HCDs in different species. Some rough rules have been demonstrated in previous studies of the HCD distribution in various animal species; for instance, almost all mammals contain both carnosine and anserine (or ophidine in whales) [17,18], amphibians contain primarily carnosine [19,20], birds contain more anserine than carnosine [7,10], and reptiles primarily contain ophidine [20,21]. In fish, the distribution patterns of HCDs in skeletal muscle revealed high concentration of HCDs in migratory pelagic fish (such as marlin, trout, tuna, and salmon) and low levels of HCDs in the families of *Pleuronectidae* (flounder) and *Percidae* (perciform fish) [22]. Considering the physiological roles of HCDs in sport and muscle functions, the high concentration of HCDs in migratory fishes may help these fishes with long distance swimming. On the other hand, some fishes, such as the families of *Clupeidae* (herrings and sardines) and *Cyprinidae* (carps), contain very high concentrations of l-histidine instead of HCDs, suggesting that the role of HCDs is replaced by l-histidine and that this therefore mainly relates to the imidazole ring of l-histidine (e.g., the pH buffering function) [22].

When considering the occurrence of the three HCDs in all investigated fish species, only *Larimichthys crocea* (small yellow fish) and *Trichiurus lepturus* (ribbon fish) contained all three HCDs in their muscles, seven species contained both carnosine and anserine, eight species contained only anserine, and three species contained only homocarnosine. In the species that contained both carnosine and anserine, the ratio of anserine/carnosine measured in this study revealed a range from 1.5 (*Larimichthys crocea*) to 504.4 (*Thunnus tonggol*). According to previous literature, most fish species contain one or two carnosine analogues in their muscle, and anserine seems to be the predominant component of HCDs. The obtained results confirmed this viewpoint, but the high proportion of anserine in fish, as well as in birds, still needs to be elucidated. None of the species contained only carnosine, but we noticed that three species contain homocarnosine in their muscle (*Scomber japonicas*, *Harpadon nehereus*, and *Anguilliformes*) with concentrations from 0.007–0.491 μg/g. Homocarnosine was reported previously as a mammal-specific HCD found mainly in the nervous system [23,24], and the exact biological function of homocarnosine in mammalian systems remains almost completely unknown. Our data revealed first that homocarnosine along with carnosine and anserine can be detected at low levels in the skeletal muscle of several fish species, indicating the possible role of homocarnosine in these species. In addition, our results also revealed that the concentration of homocarnosine in those fish species containing only this molecule is higher than that in species containing all of three HCDs, suggesting that the physiological roles of carnosine and anserine may be replaced by homocarnosine in the species.

In the present study, the concentration of HCDs in aquatic invertebrate species, including six molluscs and six crustaceans, was determined (Table 1). It is not surprising that HCDs were not detected in most of the tested invertebrate species, with the exception of *Uroteuthis chinensis*, a sepia whose dorsal muscle contains both carnosine and anserine at low levels (<0.02 μg/g). In general, HCDs have only been detected in vertebrates and not in invertebrates for now, although the presence of anserine and carnosine in Honduran prawn was reported in a peer-reviewed paper without confirmation [25] and in the whole soft body of molluscs with extremely low levels [26]. In addition, carnosine was measured previously in the whole soft body of a freshwater gastropod (*Pomacea canaliculata*) using an amino acid analyser, and a very low concentration of carnosine (0.02 nmol/mg fresh mass) was reported in this snail [27]. Considering the results from previous studies and that only muscle tissue was used for HCD detection in this study, we cannot exclude the possible presence of HCDs in the whole body of invertebrates (especially molluscs).

## 4 Conclusions

Carnosine, anserine and homocarnosine are the three most representative compounds of the HCD family and widely distributed in animals in different amounts depending on the species and tissues. This study analysed in triplicate skeletal muscle from a total of 39 species, including 35 wild aquatic species collected from the Zhoushan fishing ground and 4 commonly consumed foods species from supermarkets. The distribution pattern of HCDs in these species revealed that (i) almost all the tested fish contain HCDs, and the highest value was presented in migratory pelagic fishes; (ii) no HCD can be detected in invertebrates with the exception of the family Loliginidae; (iii) anserine is the major (or the only) component of HCDs in the tested species; (iv) only five fish species contain homocarnosine with low levels. The present findings greatly expanded the HCD distribution data and provided a clue for understanding the roles of HCDs in different animals and a nutritional assessment for marine aquatic species.

## Declaration of interests

The authors declare that they have no known competing financial interests or personal relationships that could have appeared to influence the work reported in this paper.
